# A Non-Canonical p75HER2 Signaling Pathway Underlying Trastuzumab Action and Resistance in Breast Cancer

**DOI:** 10.3390/cells13171452

**Published:** 2024-08-29

**Authors:** Babak Nami, Zhixiang Wang

**Affiliations:** Department of Medical Genetics, Faculty of Medicine and Dentistry, University of Alberta, Edmonton, AB T6G 2H7, Canada; babak.namimollalou@sickkids.ca

**Keywords:** HER2, trastuzumab, breast cancer, drug resistance, signaling pathways, regulated intramembrane proteolysis, p75HER2, ADAM10, γ-secretase

## Abstract

Overexpression of HER2 occurs in 25% of breast cancer. Targeting HER2 has proven to be an effective therapeutic strategy for HER2-positive breast cancer. While trastuzumab is the most commonly used HER2 targeting agent, which has significantly improved outcomes, the overall response rate is low. To develop novel therapies to boost trastuzumab efficacy, it is critical to identify the mechanisms underlying trastuzumab action and resistance. We recently showed that the inhibition of breast cancer cell growth by trastuzumab is not through the inhibition of HER2 canonical signaling. Here we report the identification of a novel non-canonical HER2 signaling pathway and its interference by trastuzumab. We showed that HER2 signaled through a non-canonical pathway, regulated intramembrane proteolysis (RIP). In this pathway, HER2 is first cleaved by metalloprotease ADAM10 to produce an extracellular domain (ECD) that is released and the p95HER2 that contains the transmembrane domain (TM) and intracellular domain (ICD). p95HER2, if further cleaved by an intramembrane protease, γ-secretase, produced a soluble ICD p75HER2 with nuclear localization signal (NLS). p75HER2 is phosphorylated and translocated to the nucleus. Nuclear p75HER2 promotes cell proliferation. Trastuzumab targets this non-canonical HER2 pathway via inhibition of the proteolytic cleavage of HER2 by both ADAM10 and γ-secretase. However, p75HER2 pathway also confers resistance to trastuzumab once aberrantly activated. Combination of trastuzumab with ADAM10 and γ-secretase inhibitors completely blocks p75HER2 production in both BT474 and SKBR3 cells. We concluded that HER2 signals through the RIP signaling pathway that promotes cell proliferation and is targeted by trastuzumab. The aberrant HER2 RIP signaling confers resistance to trastuzumab that could be overcome by the application of inhibitors to ADAM10 and γ-secretase.

## 1. Introduction

The family of human epidermal growth factor receptors (HERs) is composed of four members, including HER1(EGFR), HER2, HER3, and HER4 [1,2]. While the role of HER4 in breast cancer is controversial, EGFR, HER2, and HER3 are strongly implicated in breast cancer [3,4]. Overexpression of HER2 occurs in 25% of breast cancer [3,5,6,7,8,9,10]. Targeting HER2 has proven to be an effective therapeutic strategy for HER2-positive breast cancer [11,12,13,14]. Trastuzumab is the most used HER2 targeting agent, which has significantly improved patient outcomes.

Trastuzumab is a humanized recombinant monoclonal antibody to HER2, which selectively exerts antitumor effects in HER2+ breast cancer patients [13,14]. Trastuzumab binds to the HER2 extracellular juxtamembrane region. While many mechanisms have been proposed for its antitumoral activity, including both extracellular and intracellular actions, the exact mechanisms are not known and may be context dependent [13,14,15,16,17]. The extracellular action is through antibody-dependent cellular cytotoxicity (ADCC), which is well supported by various studies [18,19,20,21,22,23,24,25,26]. The data regarding the intracellular mechanisms have been controversial [16,27,28,29,30,31]. While the inhibition of canonical HER2 signaling is the basis for developing trastuzumab and has been referenced in most reviews [13,32,33], it is not supported by experimental results, including our own [13,14,15,34,35,36,37,38,39].

HER receptors are activated through homo- and hetero-dimerization [2,3,4,16,40,41] to stimulate various signaling pathways regulating multiple cell functions, which is referred to as canonical signaling [2,4,16,42]. Regulated intramembrane proteolysis (RIP) is an important non-canonical signaling strategy of many membrane receptors, including HER4 [43,44,45], TNF receptor 1 [46], and TrkA receptor [47]. This mechanism involves two proteolytic cleavage events. The first extracellular cleavage releases an ectodomain (ECD), and the second intramembrane cleavage by γ-secretase releases a soluble intracellular domain (ICD). ICD translocates to the nucleus [48] and operates as a transcriptional co-factor [49]. Both EGFR and HER2 were reported to localize to the nucleus and regulate gene transcription [50,51,52,53,54], but it is not clear whether this is through RIP.

HER2 is composed of an ECD, transmembrane domain (TM), and ICD. HER2 contains a nuclear localization signal (NLS) within its intracellular juxtamembrane region [55]. While overactivation of HER2 in breast cancer is mostly due to gene amplification, somatic mutations are present in about 4% of breast cancer patients [56]. There are many HER2 mutants with truncations and deletions. Truncated p95HER2 arises through two different mechanisms: proteolytic shedding of the ECD and alternative translation from internal initiation codons [27,57,58]. Shedding of the ECD is achieved by the metalloprotease ADAM10 at a site proximal to the transmembrane domain, generating a 95-kD membrane-anchored p95HER2 [59,60]. Alternative translation starts from internal initiation codons at positions 611 that also generate a p95HER2. Both p95HER2 forms are constitutively active [61,62] and they constitute a biomarker of an aggressive subtype of HER2+ breast cancer [63,64,65]. p95HER2 lacks the trastuzumab epitope and promotes trastuzumab resistance [29,57]. Both 75 and 42 kD HER2 fragments are detected as products of HER2 proteolytic cleavage. The 42 kD fragment was the product cleaved by calpain; however, no enzyme was identified for the 75 kD fragment [66]. The data are controversial regarding the cleavage of HER2 by γ-secretase. One study indicated that HER2 was not cleaved by γ-secretase [67], whereas another study showed the intramembrane cleavage of HER2 by γ-secretase [68].

While trastuzumab is the most used HER2 targeting agent, which has significantly improved outcomes, the overall response rate is low. Primary resistance is observed in >50% of HER2+ patients treated with trastuzumab alone. For patients who initially responded to trastuzumab, the majority eventually develop acquired resistance [69,70]. Overcoming trastuzumab resistance is a great challenge, and it is the focus of this study. To develop novel therapies to boost trastuzumab efficacy, it is critical to identify the mechanisms underlying trastuzumab action and resistance. Recently, we studied the mechanism of trastuzumab action and resistance. We found that trastuzumab did not inhibit the homo-dimerization and phosphorylation of HER2 in Chinese hamster ovarian (CHO) cells stably expressing HER2 [26,71]. Trastuzumab did not inhibit the dimerization and phosphorylation of HER2 in HER2+ BT474 and SKBR3 cells, however, trastuzumab inhibited the proliferation of these cells [72]. These results indicate that the inhibition of breast cancer cell growth by trastuzumab is not through the inhibition of HER2 canonical signaling. Here we showed that HER2 signaling also acts through the RIP pathway, which is targeted by trastuzumab. Specifically, we showed that HER2 is proteolytically cleaved extracellularly by ADAM10 and intramembranely by γ-secretase to release an intracellular p75HER2. p75HER2 is transported to the nucleus, which promotes the proliferation of breast cancer cells. Trastuzumab attenuates this p75HER2 pathway by blocking HER2 cleavage. However, aberrant activity of p75HER2 due to high proteolytic activity confers resistance to trastuzumab in breast cancer. Inhibition of this p75HER2 pathway by inhibitors to ADAM10 and γ-secretase enhanced the efficacy of trastuzumab in inhibiting P75HER2 production.

## 2. Materials and Methods

### 2.1. Cell Culture and Treatments

MCF7, MDA-MB-231, SKBR3, BT474, MCF10, and 293T cell lines were purchased from American Type Culture Collection (ATCC; Manassas, VA, USA). CHO cell lines were obtained as gifts from Dr. Luc Berthiaume (University of Alberta). CHO-HER2 cells (stably overexpressing human HER2) were obtained as gifts from Dr. Holger Buchholz (Paul-Ehrlich-Institute, Langen, Germany) [73]. The cells were cultured in Dulbecco’s modified Eagle’s medium (DMEM) medium supplemented with 10% fetal bovine serum (FBS) and antibiotics including penicillin (100 U/mL) and streptomycin (100 μg/mL) and were maintained at a 5% CO_2_ atmosphere at 37 °C. The transgenic selection was maintained by adding G418 (200 µg/mL) for CHO-HER2 to the culture medium. The cells were starved overnight (16 h) at DMEM containing 1% FBS before the treatments.

### 2.2. Chemicals and Antibodies

Vinorelbine tartrate, TAPI-2, RO-4929097, LY411575, GI254023X, and recombinant human EGF, goat anti-mouse IgG-agarose antibody, isotype human IgG, and other chemicals and reagents were purchased from Sigma-Aldrich (St. Louis, MO, USA). CP-724714 was purchased from Selleckchem (Houston, TX, USA). Pertuzumab and trastuzumab (Herceptin^®^) were purchased from Roche (Basel, Switzerland). Mouse monoclonal antibodies against HER2 N-terminus (9G6), whole HER2 (A2), α-tubulin (B-7), GFP (B-2), and rabbit polyclonal antibodies against C-terminal HER2 (C18) were purchased from Santa Cruz Biotechnology Inc. (Dallas, TX, USA). Rabbit polyclonal anti-human pY1005 and pY1139, were purchased from FroggaBio (Toronto, ON, Canada). Anti-rabbit and anti-mouse RDye^®^ 800CW and RDye^®^ 650 secondary antibodies were purchased from LI-COR Biotechnology Inc. (Lincoln, NE, USA).

### 2.3. Plasmids

GFP-tagged HER2 was previously constructed [74]. Truncated HER2 cDNA ORFs were amplified by PCR using previously constructed EGFP-N3-ERBB2 as a PCR template. The sequences of the PCR overhang oligonucleotide primers are as follows: Sense 3′-AAACTCGAGAACatgGGGATCCTCATCAAGCGACGG-5′ and antisense 3′-CCCAAGCTTCACTGGCACGTCCAGACCC3′-5′ for p75HER2; sense 3′-AAACTCGAGAAAatgGAAACGGAGCTGGTGGAGCC-5′ and antisense 3′-CCCAAGCTTCACTGGCACGTCCAGACCC3′-5′ for ΔNLSp75HER2. The PCR reaction was performed using the ACCUZYME™ DNA Polymerase Kit (Bioline; Memphis, TN, USA) following the manufacturer’s instructions. The ligation mixes were then transformed into competent E. coli by the heat shock method. The bacteria were cultured overnight on agar plates containing ampicillin. Twenty colonies were separately expanded by culturing in lysogeny broth (LB) culture, and then the plasmid contents were extracted and run in 1% agar gel by DNA electrophoresis. The colonies possessing pDrive-ERBB2 plasmids were expanded, and high-volume pDrive-ERBB2 plasmids were extracted.

ERBB2 ORFs were sub-cloned from pDrive-ERBB2 plasmids into pEGFP-N3 vectors. For this, the empty target plasmids and pDrive-ERBB2 plasmids were digested by Xho1 and HindIII restriction enzymes to provide sticky ends. The digested pDrive-ERBB2 mixture was mixed with digested pEGFP-N3 plasmids, and T4 DNA ligase was added to the mixture. All enzymes were purchased from New England Biolab (Ipswich, MA, USA). The ligation mixtures were transformed into competent E. coli, and the bacteria were cultured overnight on an agar plate containing kanamycin. After the culture, 20 colonies from each transformation were separately expanded by culture in LB overnight, and the plasmids were extracted. Successful colonies were selected based on plasmid size after electrophoresis run into 1% agarose gel and were expanded by culture to extract a high volume of pEGFP-ERBB2 plasmids. Successful cloning was confirmed by PCR amplification of ERBB2 ORFs as described above.

### 2.4. Plasmid Transfection

Approximately 10^6^ MCF7 cells were plated 24 h prior to transfection. Three hours prior to transfection, the culture medium was replaced with 0.5 mL (for 24-well culture) or 7.5 mL (for 10 cm plate culture) of antibiotic-free Opti-MEM medium. Amounts of 0.5 µL (for 24-well culture) or 5 µL (for 10 cm plate culture) of Lipofectamine^®^ 2000 (cat# 11668027; Thermo Fisher Scientific, Waltham, MA USA) were mixed in 50 µL (for 24-well culture) or 750 µL (for 10 cm culture) of Opti-MEM medium, and the lipofectamine solution was incubated at room temperature for 5 min. Approximately 1 µg (for 24-well culture) or 10 µg (for 10 cm culture) plasmid DNA was mixed in 50 µL (for 24-well culture) or 750 µL (for 10 cm culture) of Opti-MEM medium. Then, the DNA solution was added to the lipofectamine solution dropwise. The transfection mixture was then mixed by pipetting and left to incubate at room temperature for 30 min. Afterward, the transfection mixture was added to the cells drop-wise, and the cells were left to incubate for 6 h at culture conditions. After incubation, the medium was replaced with fresh DMEM medium supplemented with 10% FBS and antibiotics, including penicillin (100 U/mL) and streptomycin (100 μg/mL), and the cells were left to culture overnight.

### 2.5. Cell Proliferation (Viability) Assay by MTT

The same number of cells (10^4^) were seeded in each well containing 200 μL DMEM with 10% FBS in a 96-well plate. After culture overnight, the cells were treated with EGF and/or various agents in fresh DMEM. Cell proliferation was assessed by MTT (3-(4,5-Dimethylthiazol-2-Yl)-2,5-Diphenyltetrazolium Bromide) assay using the Vybrant MTT Cell Proliferation Assay Kit from Thermo Fisher Scientific (Waltham, MA USA) according to the manufacturer’s instructions. The cell numbers were reflected by the color intensity at 540 nm wavelength that was measured by a microplate reader.

### 2.6. Subcellular Fractionation

Proteins from membrane, cytosol, and nuclear fractions were isolated by using the Subcellular Protein Fractionation Kit (cat# NBP2-47659; Novus Biologicals, Centennial, CO, USA) following the manufacturer’s instructions. The culture medium was discarded, and the cells (5–10 × 10^6^) were washed with PBS and trypsinized. The cells were collected in a 15 mL tube and pelleted by centrifugation at 200× *g* for 5 min. The cell pellet was washed with ice-cold PBS and pelleted again by centrifugation at 200× *g* for 5 min. The pellet was resuspended in 400 µL of ice-cold Cytosol Extraction Buffer-Mix (CEB-Mix) containing 2 mM dithiothreitol (DTT) and protease inhibitor cocktail by gentle pipetting and incubated at 4 °C with rocking for 20 min. The cells were then centrifuged at 700× *g* for 10 min. The supernatant was collected in a new tube as cytosolic protein fraction. The pellet was resuspended in 400 µL of ice-cold Membrane Extraction Buffer-A Mix (MEB-A Mix) containing 2 mM DTT and protease inhibitor cocktail by vigorous vortexing for 20 s. Then, 22 µL of Membrane Extraction Buffer-B was added to the mixture and mixed by vortexing. The mixture was incubated on ice for 1 min and then centrifuged at 1000× *g* for 10 min. The supernatant was collected in a new tube as membrane protein fraction and stored at −80 °C. The pellet was resuspended in 200 µL of ice-cold Nuclear Extraction Buffer Mix (NEB-Mix) containing 2 mM DTT and protease inhibitor cocktail by vortexing and was incubated at 4 °C with rocking for 40 min. Afterward, the mixture was centrifuged at 14,000× *g* for 10 min, and then the supernatant was collected into a new tube as nuclear protein fraction and stored at −80 °C. All centrifugations were performed at 4 °C.

### 2.7. Cell Lysates and Immunoblotting

Cell lysates were prepared as previously described [26]. Briefly, the cells were collected by scraping and lysed in ice-cold Mammalian Protein Extraction Reagent (Pierce, Rockford, IL, USA) containing a protease and phosphatase inhibitor cocktail (0.02% NaN_3_, 0.1 mM 4-(2-aminoethyl)-benzenesulfonyl fluoride, 1 µM pepstatin A, 10 µg/mL aprotinin, 0.5 mM Na_3_VO_4_). Following the incubation on ice for 1 h, the cell lysates were centrifuged at 21,000× *g* for 15 min at 4 °C. The supernatant was collected, mixed with an equal volume of 2× sample buffer, and boiled for 5 min. Following gel electrophoresis, the proteins were transferred to a nitrocellulose membrane. The nitrocellulose membrane was immunoblotted with various primary antibodies as indicated. The protein bands were detected and analyzed by using the Odyssey CLx imaging system (LI-COR Biotechnology Inc., Lincoln, NE, USA) as described [75].

### 2.8. Immunofluorescence

Immunofluorescence was performed as previously described [76]. Cells (105) were seeded on 15 mm round cover glass in 24-well plates and were cultured for 48 h to allow the cells to settle and attach. Cells were treated in the same way as described in 5.1. Following the treatment, cells were washed with ice-cold PBS and then fixed with −20 °C methanol for 5 min. Afterwards, the cells were washed with TBS and blocked with TBS containing 1% bovine serum albumin (BSA) for 60 min. Cells were incubated with 2 µg/mL of primary antibodies as indicated for 60 min. The cells were washed and then incubated with 1 µg/mL FITC-conjugated and/or 1 µg/mL rhodamine-conjugated secondary antibodies for 60 min in dark. Afterwards, the cells were washed with TBS and then incubated in 1 µg/mL DAPI solution for 5 min. The coverslips were mounted on microscope slides and examined with a deconvolution microscope system (GE Healthcare Life Science, Mississauga, ON, Canada). All of the images were deconvolved. The selected images were line scanned with the software SoftWoRx 7.0 embedded in the microscope system. The line scan measures the intrinsic intensity of individual fluorescence channels and will not be affected by changing the contrast and brightness.

## 3. Results

### 3.1. Trastuzumab Specifically Binds to HER2

**Figure 1:**
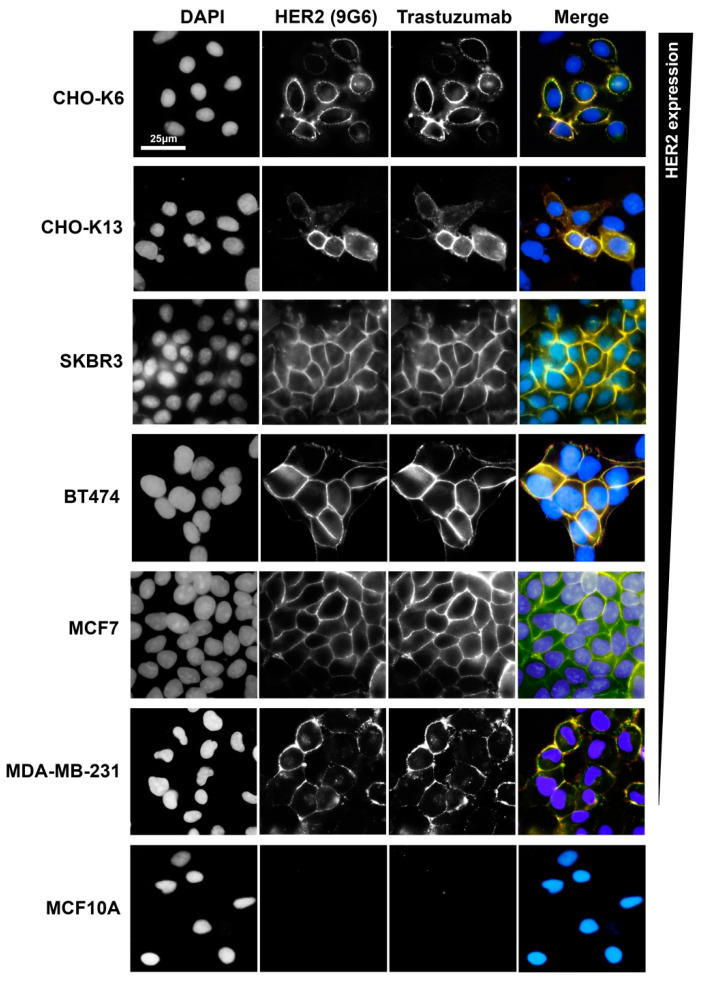
Trastuzumab binding to HER2 in breast cancer cell lines. Binding of trastuzumab to HER2 in two CHO cell lines (CHO-K6 and CHO-K13) expressing HER2, breast cancer cell lines with high HER2 expression levels (SKBR3 and BT474), low HER2 expression levels (MCF7 and MDA-MB-231), and HER2-negative breast cell line (MCF10). The cells were treated with 10 μg/mL trastuzumab for 1 h. HER2 was stained by mouse monoclonal antibody 9G6, followed by FITC (green)-conjugated anti-mouse IgG. Trastuzumab was stained by TRITC (red)-conjugated anti-human IgG. Scale bar: 25 μm. The gradient bar indicates HER2 expression level.

We showed previously that trastuzumab only specifically binds to HER2, not other HER receptors, including HER1 (EGFR) and HER3 [26]. Here, we examined if trastuzumab binds to HER2-positive breast cancer cell lines including SKBR3, BT474, MCF7, and MDA-MB-231 by double immunofluorescence staining of HER2 and trastuzumab (Figure 1). CHO-HER2 stable cell lines ectopically expressing HER2, including CHO-K6 and CHO-K13, were used as positive controls [26,73]. MCF10A cells that do not express HER2 were used as a negative control. Results showed very specific co-localization of trastuzumab and HER2 in HER2-expressing cells, but not in HER2-negative cells, which indicates that trastuzumab binds to HER2 in these breast cancer cells (Figure 1).

### 3.2. Trastuzumab Inhibits Proliferation of HER2-Positive Breast Cancer Cells

**Figure 2:**
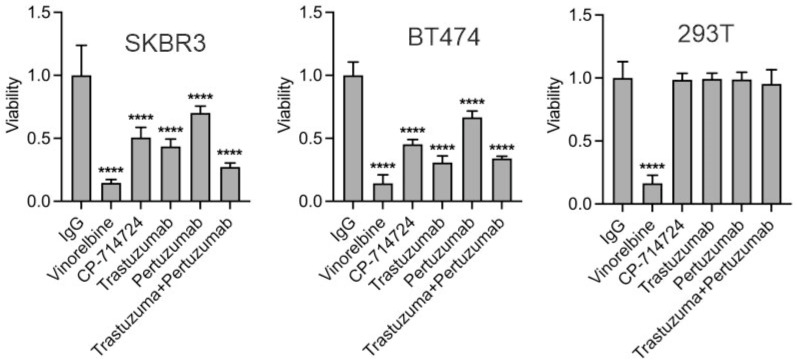
Trastuzumab inhibits the proliferation of HER2-positive breast cancer cell lines. SKBR3, BT474, and 293T cells were treated with 10 μg/mL trastuzumab, pertuzumab, or their combination for 5 days, and then the cell proliferation was evaluated by MTT assay (absorbance at 540-nanometer wavelengths). Ten micrograms/millilitres human IgG, 10 µM vinorelbine, and 10 μM CP-724714 were used as respectively mock, anti-proliferative, and HER2 inhibitor controls. ****: *p* < 0.0001.

To investigate whether binding of trastuzumab to HER2 inhibits the proliferation of HER2-positive breast cancer cells, we treated SKBR3 and BT474 cell lines with 10 μg/mL trastuzumab for 5 days and monitored the cell proliferation levels by MTT assay. Cells treated with non-specific human IgG (10 μg/mL), cell cycle inhibitor vinorelbine (10 μM), and HER2 kinase inhibitor CP-714724 (10 μM) were used as mock control, anti-proliferative control, and HER2 inhibition control, respectively. We also tested the effect of pertuzumab (10 μg/mL) alone as well as in combination with trastuzumab. 293T cells were used as HER2-negative cell control. As shown in Figure 2**,** trastuzumab (*p* < 0.0001) as well as other HER2-targeting agents CP-714724 (*p* < 0.0001) and pertuzumab (*p* < 0.0001) significantly inhibited the proliferation of SKBR3 and BT474 cells but not 293T cells. These results show that the binding of trastuzumab to HER2 inhibits cell proliferation of HER2-positive breast cancer cell lines, probably via blocking HER2 function.

### 3.3. Proteolytic Cleavage of HER2 and Its Inhibition by Trastuzumab

We showed above that trastuzumab inhibits cell proliferation. We previously showed that trastuzumab did not inhibit the dimerization and phosphorylation of HER2 in HER2+ BT474 and SKBR3 cells [72]. We also showed that trastuzumab inhibited the cell proliferation without inhibiting the homo-dimerization and phosphorylation of HER2 in CHO cells stably expressing HER2 [26,71]. These results indicate that the inhibition of breast cancer cell growth by trastuzumab is not through the inhibition of HER2 canonical signaling. There may be a novel non-canonical HER2 pathway that is targeted by trastuzumab.

**Figure 3:**
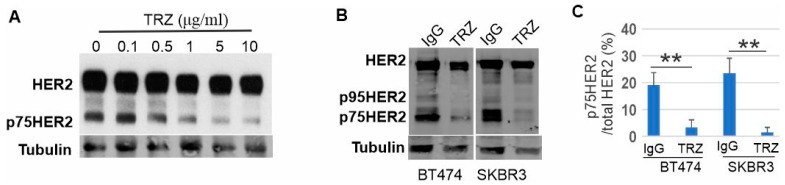
Proteolytic cleavage of HER2 and its inhibition by trastuzumab (**A**) CHO-HER2 cells were treated with EGF and trastuzumab with various concentrations (0, 0.1, 0.5, 1, 5, and 10 μg/mL), and the cell lysates were immunoblotted with anti-HER2 antibody (A2). (**B**) BT474 and SKBR3 cells were treated with trastuzumab (10 μg/mL) or normal human IgG (μg/mL), and the cell lysates were immunoblotted with anti-HER2 antibody (A2). (**C**) the quantification of the data from (**B**). ** *p* < 0.01.

Indeed, we observed a truncated band with an approximate size of 75 kD (p75HER2) in immunoblotting of CHO-HER2 cell lysates with a polyclonal anti-HER2 antibody A2, which suggests that HER2 is proteolytically cleaved (Figure 3A). Interestingly, treatment with trastuzumab blocked the production of the truncated band in a dose-dependent manner (Figure 3A). Polyclonal anti-HER2 antibody A2 recognizes both N- and C-terminal HER2. To further explore this observation, we treated SKBR3 and BT474 cells with trastuzumab (10 μg/mL) and normal human IgG (10 μg/mL) each for 6 h and then immunoblotted HER2 using another antibody specific against the C-terminal end of HER2 (C18). As shown in Figure 3B,C, there were several truncated HER2 fragments with sizes smaller than 185 kD in both cell lines treated with normal human IgG. The most prominent band has a molecular mass close to 75 kD. We name this truncated fragment as p75HER2. The most reported p95HER2 [27,57,58] was also visible in the blot. Treatment with trastuzumab significantly decreased levels of both p75HER2 and p95 HER2 in SKBR3 and BT474 cell lines (Figure 3B,C). These data indicated that HER2 is proteolytically cleaved and trastuzumab inhibits the cleavage of HER2.

### 3.4. p75HER2 Is Cytosolic and Translocated to Nucleus

**Figure 4:**
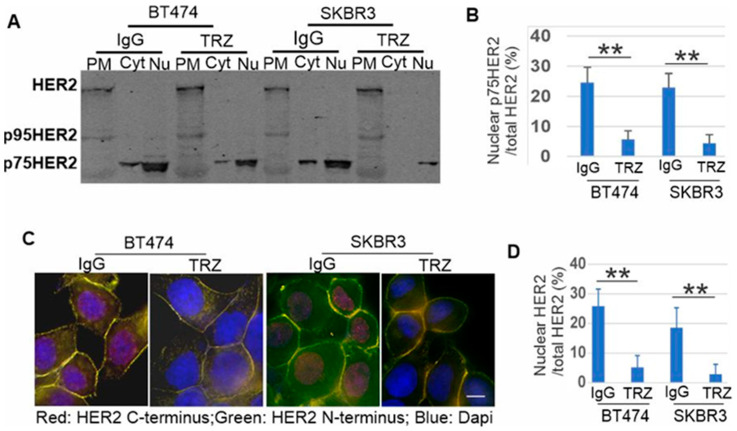
Nuclear localization of p75HER2 and its inhibition by trastuzumab. (**A**) Subcellular fractionation to show the nuclear p75HER with or without trastuzumab (10 μg/mL). HER2 was detected by antibody to HER2 (A2). (**B**) Quantification of the data from A. We loaded 1/10th of the total proteins isolated from the plasma membrane (PM) and cytosolic (Cyt) fraction, but 1/4th of the proteins isolated from nucleus. We normalized this in our quantification. (**C**) Nuclear localization of HER2 C-terminus by immunofluorescence. Cells were double stained with antibodies to HER2 N-(9G6, green) and C-terminus (c-18, red) and counter stained with Dapi (blue). Size bar = 10 μm. (**D**) Quantification of the data from C. C-18 stain (red) is positive for both full length and p75HER2 and is used for quantification. The nuclear localization of HER2 was expressed as the percentage of nuclear intensity out of the total cell intensity. ** *p* < 0.01.

Our results in Figure 3 are consistent with a previous report that HER2 is proteolytically cleaved to generate an intracellular p75HER2 [66]. It is also shown recently that β2-AR activation promotes cleavage and nuclear translocation of HER2 [68]. We next determined if p75HER2 is HER2 ICD and is translocated to the nucleus. The homogenates of both BT474 and SKBR3 cells were subcellular fractionated into the plasma membrane (PM), cytosolic (Cyt), and nuclear (Nu) fractions and lysed. Immunoblotting with anti-HER2 antibody (A2) showed that in both BT474 and SKBR3 cells, the full-length HER2 is only presented in the plasma membrane fraction; p75HER2 is mostly in the nuclear fraction, but also in the cytosolic fraction. The p75HER2 accounts for more than 20% of the total HER2 (Figure 4A,B). Treatment with trastuzumab significantly reduced both cytosolic and nuclear p75HER2, and the p75HER2 accounts for less than 5% of the total HER2 (Figure 4A,B). We also observed p95HER2 in the PM fraction.

We then examined by immunofluorescence if p75HER2 HER2 ICD and is translocated to the nucleus. We stained BT474 and SKBR3 cells with two HER2 antibodies, one specific for the N-terminal HER2 (9G6) and one specific for the C-terminal HER2 (C18). We showed that N-terminal HER2 is only localized to the PM, but C-terminal HER2 is localized to both the PM and the nucleus (Figure 4C,D), which indicates that the nuclear HER2 is the truncated C-terminal HER2 ICD. Treatment with trastuzumab strongly inhibited the nuclear stain of the C-terminal truncated HER2 (Figure 4C,D). Quantification of the stain showed that nuclear C-terminal HER2 accounts for approximately 20% of the total HER2 in the control; however, treatment with trastuzumab reduced the percentage to less than 5% (Figure 4C,D), which is consistent with the results of subcellular fractionation.

These results indicate that p75HER2 is the cleaved HER2 ICD. Following the cleavage, p75HER2 is translocated from the cytoplasm to the nucleus. Trastuzumab strongly reduces nuclear HER2 by inhibiting the cleavage of p75HER2.

### 3.5. Phosphorylation of p75HER2 and the Effects of Trastuzumab

**Figure 5:**
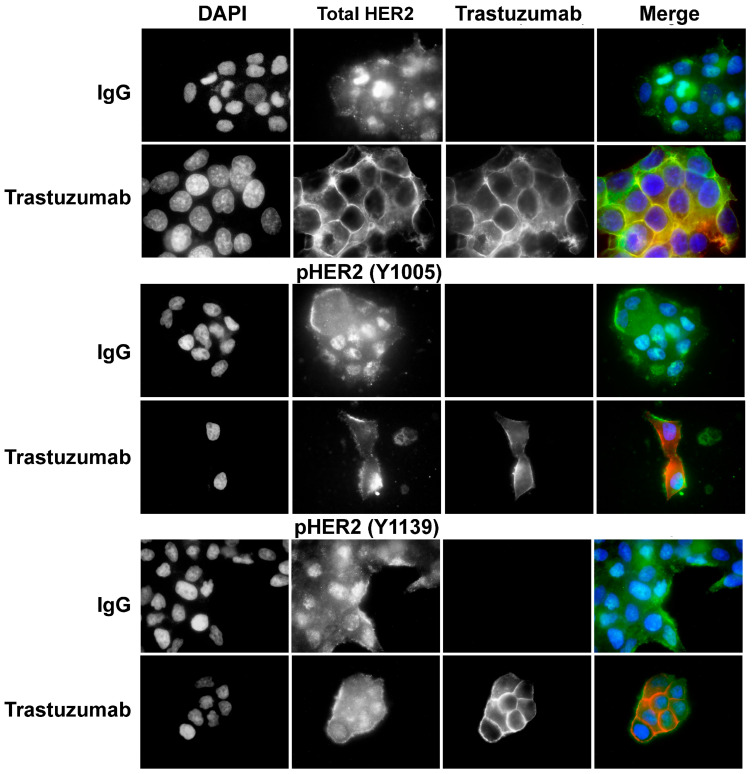
Phosphorylation of p75HER2 and the effects of trastuzumab on BT474 cells were treated with trastuzumab (10 μg/mL) or normal human IgG (10 μg/mL). The phosphorylation and localization of HER2 were examined by immunofluorescence with antibodies to HER2, pY1005 HER2, and pY1139 HER2. Scale bar: 20 μm.

We next examined the phosphorylation status of p75HER2 by immunofluorescence in BT474 cells. We used two antibodies, one specific for Y1005 phosphorylation (pY1005) and one specific for Y1139 phosphorylation (pY1139). We showed that nuclear localized p75HER2 is stained positive for both pY1005 and pY1139, which suggests that the nuclear localized p75HER2 is phosphorylated. Treatment with trastuzumab significantly reduced the nuclear stain of both pY1005 and pY1139, which is likely due to its inhibition on HER2 cleavage to produce p75HER2 (Figure 5).

### 3.6. Generation of p75HER2 by Two Proteolytic Cleavages and Its Inhibition by Trastuzumab

**Figure 6:**
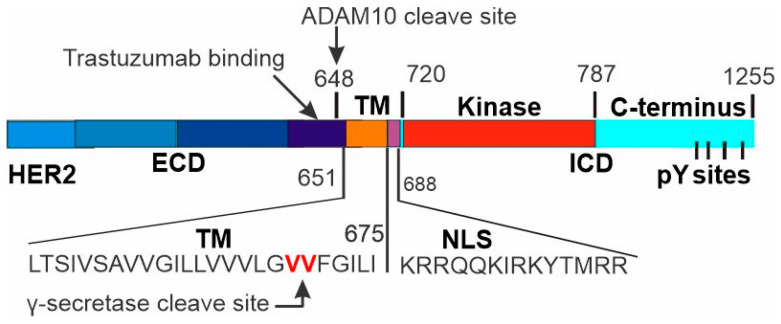
Domain structure of HER2 and the proteolytic cleavage sites.

Among HER receptors, HER4 is known to signal through the RIP pathway [43,44,45]. This mechanism involves two proteolytic cleavage events. The first extracellular cleavage releases an ECD, and the second intramembrane cleavage by γ-secretase releases a soluble ICD. ICD translocates to the nucleus [48] and operates as a transcriptional co-factor [49]. We speculate that HER2 may also signal through the RIP pathway. Indeed, it is known that HER2 is cleaved extracellularly by ADAM10 at A648, which produces two truncated HER2, the ECD that is released, and a membrane-anchored p95HER2 that contains HER2 TM and ICD (Figure 6) [59,60]. p95HER2 lacks the trastuzumab epitope and promotes trastuzumab resistance [29,57]. While it is controversial regarding the intramembrane cleavage of HER2 by γ-secretase [67,68], HER2 contains two consensus valines (V669 and V670) for γ-secretase cleavage. HER2 contains an NLS within its intracellular juxtamembrane region (Figure 6) [55,77].

**Figure 7:**
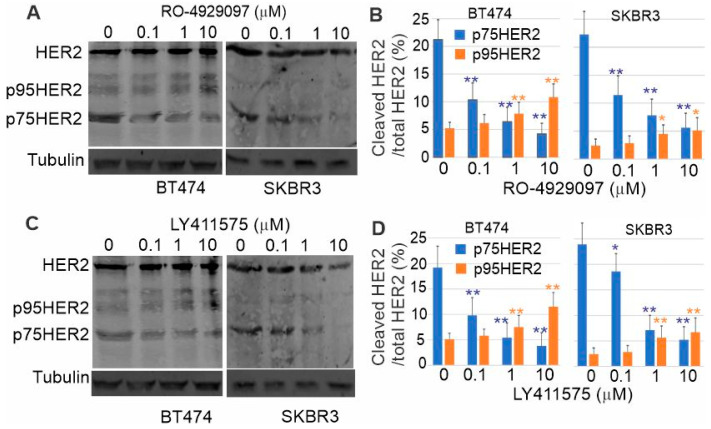
Inhibition of γ-secretase by chemical inhibitor reduces the production of p75HER2 and increase the amount of p95HER2. (**A**) Inhibition by RO-4929097. (**B**) Quantification of the data from A. (**C**) Inhibition by Ly411575. (**D**) Quantification of the data from (**C**). Each data point is the average of at least 3 repeats. *: *p* < 0.1; **: *p* < 0.01.

To determine if HER2 signals through the RIP pathway, we first determined if p75HER2 is generated by two proteolytic cleavages of the full-length HER2. We inhibited γ-secretase in BT474 and SKBR3 cells by the chemical inhibitors RO-4929097 and LY411575 and then examined the production of p75HER2 by immunoblotting. As shown in Figure 7, the p75HER2 bands were significantly reduced following the inhibition of γ-secretase, suggesting strongly that HER2 is cleaved by γ-secretase. Moreover, inhibition of γ-secretase increased the level of p95HER2 in BT474 cells (Figure 7), which suggests that p75HER is generated by dual proteolytic cleavage (Figure 7). The first cleavage generates p95HER2, which is then cleaved by γ-secretase to produce p75HER2.

**Figure 8:**
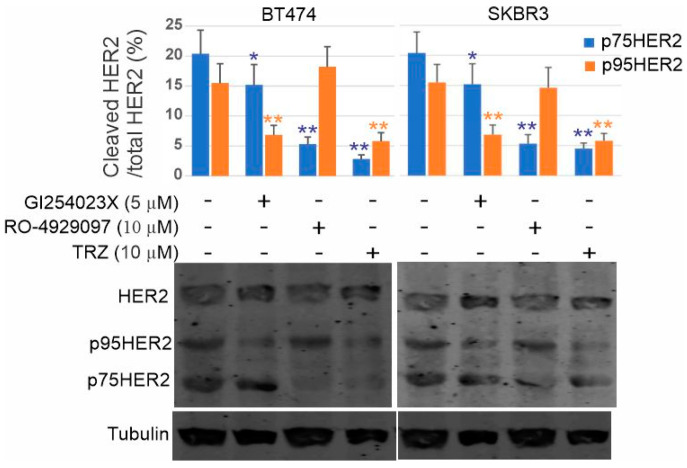
Effects of γ-secretase inhibitor RO-cj4929097, ADAM10 inhibitor G1254023X, and trastuzumab on the cleavage of HER2 to produce p75HER2 and p95HER2 in BT474 and SKBR3 cells. * *p* < 0.1, ** *p* < 0.01.

We then examined the effects of ADAM10 inhibitor and trastuzumab on the production of p75HER2. We treated BT474 cells with ADAM10 inhibitor GI254023X, γ-secretase inhibitor RO-4929097, and trastuzumab. We showed that ADAM10 inhibitor GI254023X not only strongly inhibited the production of p95HER2, but also partially inhibited the production of p75HER2. However, the γ-secretase inhibitor RO-4929097 only blocked the production of p75HER2, not p95HER2 (Figure 8). These data further indicate that p75HER2 is the product of dual cleavage of HER2 by ADAM10 and γ-secretase. Interestingly, trastuzumab blocked the production of both p95HER2 and p75HER2 (Figure 8). Our data suggest that trastuzumab inhibits the production of p75HER2 by blocking the action of both ADAM10 and γ-secretase.

### 3.7. The Role of p75HER2 in Cell Proliferation and in Trastuzumab Resistance

**Figure 9:**
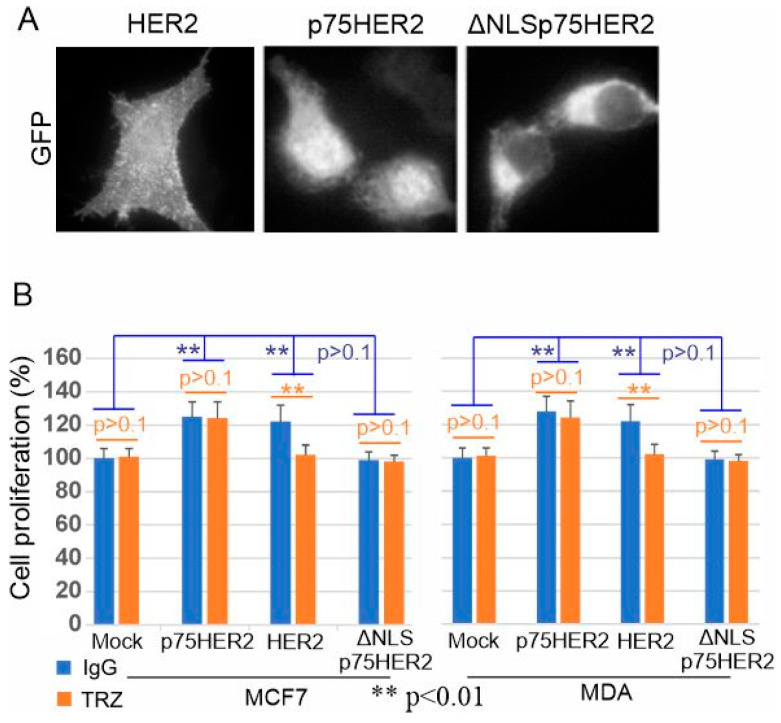
p75HER2 is mitogenic. (**A**) subcellular localization of GFP-tagged p75HER2, HER2, and ΔNLSp75HER2 in MCF-7 cells by fluorescence microscopy. (**B**) MDA and MCF-7 cells were transfected with GFP-tagged p75HER2, HER2, and ΔNLSp75HER2. The cell proliferation with or without trastuzumab (10 μg/mL) treatment was revealed by MTT assay.

To examine if the inhibitory effects of trastuzumab on the proliferation of HER2+ breast cancer cells are due to the inhibition of the non-canonical HER2 RIP pathway, we constructed GFP-tagged p75 HER2 (671–1255 aa), and ΔNLS p75HER2 (689–1255 aa, p75HER2 with the deletion of NLS) (Figure 6). The full-length HER2 was constructed previously. When expressed in MCF7 cells, GFP-tagged p75HER2 is mostly in the nucleus, the full-length HER2 in the PM, and ΔNLSp75HER2 in the cytosol (Figure 9). We then examined the effects of these HER2 constructs on the proliferation of MCF-7 and MDA-231 cells with or without trastuzumab treatment. We chose MCF-7 and MDA-231 cells due to the very low expression of endogenous HER2 in these two cell lines. We showed that both GFP-tagged HER2 and p75HER2 increased cell proliferation, but not ΔNLS p75HER2. Moreover, trastuzumab inhibits HER2-induced cell proliferation, but not p75HER2-induced cell proliferation (Figure 9). These results suggest that nuclear localized p75HER2 is mitogenic and confers resistance to trastuzumab.

### 3.8. Inhibition of p75HER2 by the Combination of Trastuzumab and Inhibitors to ADAM10 and γ-Secretase

**Figure 10:**
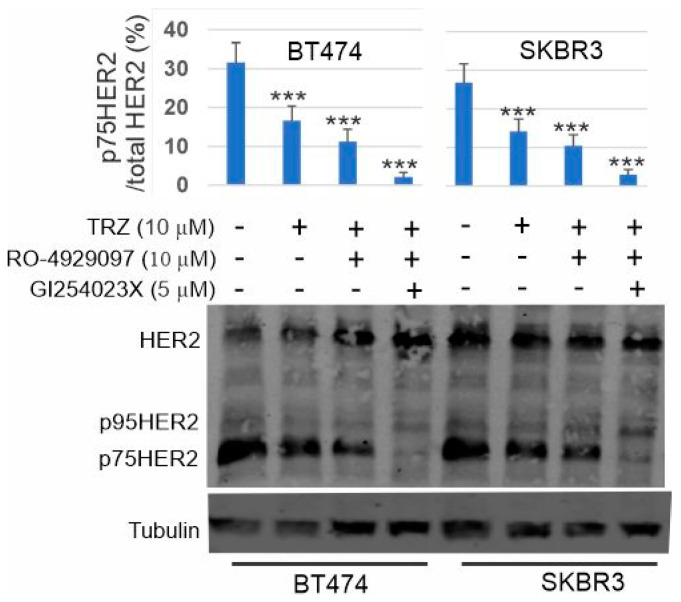
Inhibition of p75HER2 by the combination of GI254023X (5 µM), RO=4929097 (10 µM), and trastuzumab (10 µM) in BT474 and SKBR3 cells. ***, *p* < 0.001.

Our results suggest that one mechanism underlying the inhibition of cell proliferation by trastuzumab is to inhibit the production of p75HER2. Here, we examined if the additional inhibitor to ADAM10 and γ-secretase will enhance the efficacy of trastuzumab on p75HER2 production in breast cancer cells. Indeed, we showed that applying the γ-secretase inhibitor RO-4929097 significantly enhanced the effects of trastuzumab on the inhibition of p75HER2 production. Applying both ADAM10 inhibitor G1254023X and γ-secretase inhibitor RO-4929097 together with trastuzumab resulted in the complete block of p75HER2 production (Figure 10).

## 4. Discussion

Accumulated evidence suggests that the inhibitory effects of trastuzumab on breast cancer cell proliferation are not through the inhibition of HER2 dimerization and phosphorylation [26,71]. We have shown recently that trastuzumab inhibits HER2 signaling by blocking its localization to the lipid raft [72]. Here, we demonstrated that HER2 signals through a novel non-canonic signaling pathway, RIR, which is targeted by trastuzumab.

RIP is a well-established signaling pathway utilized by several membrane receptors, including TNF receptor 1 [46] and TrkA receptor [47]. Among HER receptors, only HER4 is known to signal through RIP [43,44,45]. Both EGFR and HER2 were reported to localize to the nucleus and regulate gene transcription [50,51,52,53,54], but it is not clear whether this is through RIP. While most studies do not specifically determine if the nuclear EGFR/HER2 is the cleaved soluble intracellular domain or the intact transmembrane protein, the authors tend to suggest that nuclear EGFR/HER2 is the full-length transmembrane protein. One recent article claims that the intact HER2/HER3 dimer functions inside the nucleus [78]. However, there is no demonstrated mechanism to support the translocation of a transmembrane receptor into the nucleus.

Here, we observed the presence of a truncated HER2 fragment with a molecular weight of 75 kD. This p75HER2 is present in various cell lines, including CHO-HER2 cells, BT474 cells, and SKBR3 cells. Treatment with trastuzumab significantly reduced the amount of this p75HER2 (Figure 3A), suggesting that trastuzumab interferes with the signaling pathway leading to the formation of p75HER2. This p75HER2 is distinct from the well-established p95HER2, as we observed the presence of both p95HER2 and p75HER2. Our observation is consistent with a previous study that showed that HER2 is proteolytically cleaved to generate an intracellular p75HER2 [66].

We then showed by both subcellular fractionation and immunofluorescence that p75HER2 is mostly presented in the nucleus, with some presence in the cytoplasm (Figure 4). The full-length HER2 and p95HER2 are only present in the plasma membrane. To determine if the p75HER2 is the C-terminal intracellular domain, we used three anti-HER2 antibodies with different specificities. A2 is a rabbit polyclonal antibody that recognizes both the HER2 N-terminus and C-terminus. 9G6 only reacts with the HER2 N-terminus, and C18 only reacts with the HER2 C-terminus. As shown in Figure 4B, while the 9G6 antibody only recognizes the membrane-localized HER2, the C18 antibody recognizes both the membrane and nuclear-localized HER2. These data suggest that p75HER2 is the cleaved C-terminal ICD. As p75HER2 is localized to the nucleus, it must contain the NLS that is located in the intracellular juxtamembrane region of HER2 (Figure 6).

While the data regarding the cleavage of HER2 by γ-secretase are controversial, HER2 does contain two consensus valines (V669 and V670) for γ-secretase cleavage. If HER2 is cleaved by γ-secretase, the produced ICD should be soluble with a size of 75 kD and contain NLS. Indeed, we showed that p75HER2 is the product of cleavage by γ-secretase. Treatment of the cells with either of the two γ-secretase inhibitors RO-4929097 and LY411575 blocked the production of p75HER2 in a dose-dependent manner (Figure 7).

HER2 is known to be extracellularly cleaved by metalloprotease ADAM10 to produce an ECD that is released and p95HER2 that contains TM and ICD and localized to the plasma membrane [26]. We suspect that p75HER2 is also the product of two cleavages, the first by ADAM10 to generate p95HER2, which is further cleaved by γ-secretase. To test this, we treated BT474 and SKBR3 cells with ADAM10 inhibitor GI254023X and showed that GI254023X not only strongly inhibited the production of p95HER2, but also partially inhibited the production of p75HER2. However, the γ-secretase inhibitor RO-4929097 only blocked the production of p75HER2, not p95HER2 (Figure 8). As we showed that p75HER2 is localized to the nucleus, our data support that HER2 also signals through the RIP pathway.

Indeed, we further showed that p75HER2 is phosphorylated (Figure 5) and promotes cell proliferation (Figure 9). We constructed GFP-tagged p75HER2 and ΔNLSp75HER2. (Figure 6). When expressed in MCF7 cells, GFP-tagged p75HER2 is mostly in the nucleus (Figure 9). Moreover, when expressed in MCF-7 and MDA 231 cells, GFP-tagged p75HER2 increased cell proliferation. Interestingly, ΔNLSp75HER2 was restricted to the cytoplasm and did not promote cell proliferation, which suggests that the nuclear localization is critical for the mitogenic effects of p75HER2. It is not clear how the nuclear localization of p75HER2 promotes cell proliferation. However, HER2 was shown to regulate gene transcription by acting as transcription factors (TFs) or cofactors [52,54,79,80]. As HER2 has no apparent DNA binding motif, it is possible that nuclear p75HER2 promotes cell proliferation by regulating transcription through binding to TF as a co-activator. Further research is needed to explore this possibility.

Another significant and interesting finding of this study is the inhibitory effects of trastuzumab on this novel HER2 RIP pathway. We showed that trastuzumab inhibits the cleavage of HER2 to produce p75HER2 in HER2+ breast cancer cell lines as well as CHO cells stably expressing human HER2 (Figure 3). Trastuzumab inhibits the nuclear accumulation of p75HER2 (Figure 4). Trastuzumab inhibits the nuclear accumulation of phosphorylated p75HER2 (Figure 5). Trastuzumab blocks the production of both p95HER2 and p75HER2 (Figure 8). Trastuzumab inhibits the proliferation of HER2+ breast cancer cells (Figure 2) but does not inhibit p75HER2-induced cell proliferation (Figure 9). These data suggest the inhibitory effect of trastuzumab on cell proliferation may be partly due to its inhibition of the HER2 RIP signaling pathway.

Trastuzumab inhibits the HER2 RIP pathway by interfering with the cleavage of HER2 by both ADAM10 and γ-secretase. The HER2 binding sites to trastuzumab and ADAM 10 are overlapped at the extracellular juxtamembrane region (Figure 6). The binging of trastuzumab blocks the binding of ADAM10. However, once HER2 is cleaved by ADAM10, the resulting p95HER2 lacks a trastuzumab binding site and is resistant to trastuzumab. The γ-secretase component nicastrin acts sterically to block substrates with large ectodomains from interacting with γ-secretase, providing the mechanism by which γ-secretase selectively recruits and cleaves ectodomain-shed substrates [81]. Extracellular cleavage of HER4, TrkA, and TNF receptors is required for their second intramembrane cleavage by γ-secretase. Thus, it is likely that the extracellular cleavage of HER2 by ADAM10 facilitates its intramembrane cleavage by γ-secretase. Indeed, our results showed that ADAM10 inhibitor GI254023X not only strongly inhibited the production of p95HER2, but also partially inhibited the production of p75HER2 (Figure 10). Trastuzumab may block the action of γ-secretase by two mechanisms. First, trastuzumab blocks the formation of p95HER2, which is easier accessible by γ-secretase. Second, binding of trastuzumab to HER2 will sterically further hinder the access of HER2 by γ-secretase.

While trastuzumab inhibits p75HER2 production, aberrant activity of p75HER2 due to high proteolytic activity confers resistance to trastuzumab in breast cancer. As we showed in Figure 9, once it is produced, p75HER2 promotes cell proliferation, which is resistant to trastuzumab. One way to overcome this resistance is to inhibit the production of p75HER2 by inhibiting ADAM10 and γ-secretase with inhibitors. Indeed, we showed that applying both ADAM10 inhibitor G1254023X and γ-secretase inhibitor RO-4929097 together with trastuzumab resulted in the complete block of p75HER2 production in both BT474 and SKBR3 cells (Figure 10).

## 5. Conclusions

Beside signaling through the canonical pathways, HER2 also signals through a non-canonical pathway, RIP. In this pathway, HER2 is first cleaved by metalloprotease ADAM10 to produce an ECD that is released and the p95HER2 that contains TM and ICD. p95HER2 is further cleaved by an intramembrane protease, γ-secretase, which produces a soluble ICD p75HER2 with NLS. p75HER2 is phosphorylated and translocated to the nucleus. Nuclear p75HER2 promotes cell proliferation, which is resistance to trastuzumab. Trastuzumab targets this non-canonical HER2 pathway via inhibition of the proteolytic cleavage of HER2 by both ADAM10 and γ-secretase. However, the p75HER2 pathway also confers resistance to trastuzumab once it is aberrantly activated. Combination of trastuzumab with ADAM10 and γ-secretase inhibitors completely blocks p75HER2 production in both BT474 and SKBR3 cells.

## Data Availability

The data presented in this study are available on request from the corresponding author.
